# Turnover of BRCA1 Involves in Radiation-Induced Apoptosis

**DOI:** 10.1371/journal.pone.0014484

**Published:** 2010-12-31

**Authors:** Weijun Liu, Wenjun Zong, George Wu, Takeo Fujita, Wenqi Li, Judy Wu, Yong Wan

**Affiliations:** Department of Cell Biology and Physiology, University of Pittsburgh School of Medicine and University of Pittsburgh Cancer Institute, Pittsburgh, Pennsylvania, United States of America; Wayne State University, United States of America

## Abstract

**Background:**

Germ-line mutations of the breast cancer susceptibility gene-1 (BRCA1) increase the susceptibility to tumorigenesis. The function of BRCA1 is to regulate critical cellular processes, including cell cycle progression, genomic integrity, and apoptosis. Studies on the regulation of BRCA1 have focused intensely on transcription and phosphorylation mechanisms. Proteolytic regulation of BRCA1 in response to stress signaling remains largely unknown. The manuscript identified a novel mechanism by which BRCA1 is regulated by the ubiquitin-dependent degradation in response to ionization.

**Methodology/Principal Findings:**

Here, we report that severe ionization triggers rapid degradation of BRCA1, which in turn results in the activation of apoptosis. Ionization-induced BRCA1 turnover is mediated via an ubiquitin-proteasomal pathway. The stabilization of BRCA1 significantly delays the onset of ionization-induced apoptosis. We have mapped the essential region on BRCA1, which mediates its proteolysis in response to ionization. Moreover, we have demonstrated that BRCA1 protein is most sensitive to degradation when ionization occurs during G2/M and S phase.

**Conclusions/Significance:**

Our results suggest that ubiquitin-proteasome plays an important role in regulating BRCA1 during genotoxic stress. Proteolytic regulation of BRCA1 involves in ionization-induced apoptosis.

## Introduction

Germ-line mutations in BRCA1 gene increase the susceptibility for the development of familial breast and ovarian cancers, indicating that BRCA1 functions as a tumor suppressor whose impaired activity would contribute to tumorigenesis [1]. BRCA1 has been implicated in numerous cellular processes, including DNA repair, mRNA transcription, cell cycle regulation, chromatin remodeling and protein ubiquitylation [2]. Since all these processes are involved in the maintenance of genomic stability, BRCA1 has been implicated as a key regulator of the cellular response to DNA damage. Consistent with its involvement in multiple cellular processes, BRCA1 has been shown to interact with both DNA and cellular proteins, although the exact biological function of BRCA1 remains to be defined [3], [4], [5], [6]. So far, the only known biochemical function of BRCA1 is its E3 ligase activity when BRCA1 forms a heterodimer with BARD1. Both of them have a RING-finger motif near their amino termini [7], [8], [9]. Importantly, tumor-associated mutation in the RING-finger domain of BRCA1 abolishes the ubiquitin ligase activity of the BRCA1/BARD1 complex, suggesting a strong connection between BRCA1's E3 ligase activity and its tumor suppressor function [10], [11].

Modulation of BRCA1 activity is important since any deficiency in BRCA1 activity may predispose cells to enter tumorigenesis. BRCA1 has been reported to be phosphorylated in a cell cycle dependent manner [12], [13] and also in response to ionizing radiation [14], [15]. However, the functional consequences of the phosphorylation of BRCA1 remain unclear. Speculation exists that BRCA1 phosphorylation may affect its cellular localization and stability as well as altering its ability to bind other proteins and thus, affect its biochemical activities as they are related to DNA damage repair or gene transcription [16]. Another way to modulate the activity of BRCA1 is through post-translational modifications such as ubiquitylation or sumoylation. BRCA1 has been reported to be degraded through the ubiquitin-proteasome mediated pathway [17], [18]. Moreover, the levels of protein expression for BRCA1 fluctuate during the cell cycle and this fluctuation has been demonstrated to be mediated in part by ubiquitin-proteasomal degradation [19]. Although the E3 ligase that targets BRCA1 for proteolysis remains unknown, the enhanced degradation of BRCA1 by a deregulated E3 ligase could be one of the mechanisms by which BRCA1 levels are reduced in sporadic breast cancer [20], [21]. In addition, BRCA1 can associate with Ubc9, a mediator of the conjugating ubiquitin-like protein SUMO1, suggesting that BRCA1 is susceptible to sumolynation, which may either protect the protein from degradation or affect its cellular localization [16], [22].

Previous studies have established the critical role of ubiquitylation in DNA damage response. In response to DNA damage, many proteins that are involved in checkpoint activation (e.g., Cdc25A and Chk1), chromatin remodeling (e.g., H2A, H2AX), DNA repair (e.g., FANCD2) and apoptosis regulation (e.g., Bcl-2s and IAPs) have been reported to be poly- or mono-ubiquitylated resulting in their degradation or activation as signal transducer [23], [24], [25], [26], [27], [28], [29]. BRCA1 is thought to be one of the E3 ligases responsible for DNA damage induced-ubiquitylation based on the co-localization of conjugated ubiquitin with BRCA1/BARD1 [23], [30]. Although BRCA1/BARD1 is able to ubiquitylate a number of potential targets *in vitro*, including FANCD2, RNA polymerase II, nucleoplasmin, p53, H2AX and histones H2A, H2B, H3 and H4, its *bone fide* substrates *in vivo* remain unknown [31], [32], [33]. To further understand the role of ubiquitin-proteasomal system (UPS) in genomic integrity, we have established a system to screen for degraded proteins induced by γ irradiation. Surprisingly, we found that BRCA1 is degraded in an ubiquitin-proteasome dependent manner in response to high dosage (20 Gy) γ irradiation. Our results further suggest that proteolytic regulation of BRCA1 is involved in γ irradiation-induced apoptosis.

## Materials and Methods

### Plasmids and Constructs

A set of BRCA1 mutants were engineered by PCR using the following primers:

BRCA1(70–1863) F: 5′AGGAGCCTACAAGAAAGT3′

BRCA1(367–1863) F: 5′GAAGATGTTCCTTGG ATA3′

BRCA1(1068–1863) F: 5′CAAGCAGAACTAGGTAGA3′

BRCA1(1863)R: 5′GTAGTGGCTGTGGGGGAT3′

BRCA1(1) F: 5′ATGGATTTATCTGCTCTTCGC3′

BRCA1 (1–1580) R: 5′AGAAGGATCAGATTCAGG3′

BRCA1 (1–1477) R: 5′ACTATCTGCAGACACCTC3′

BRCA1 (1–1419) R: 5′CTGTTCTAACACAGCTTC3′

BRCA1(1–1017) R: 5′TGCTTGAATGTTTTCATC3′

and then were cloned into pCS2-HA, a mammalian expression vector.

### Cell culture

HeLa cells (ATCC) or mouse embryonic fibroblast BRCA1^+/+^ and BRCA1^−/−^ (ATCC) were cultured in Dulbecco's modified essential medium (Gibco-BRL) supplemented with 10% or 15% fetal bovine serum and antibiotics. Human breast cancer cell line MCF7 (ATCC) was grown in RPMI 1640 supplemented with 10% FBS and antibiotics. All cell lines were maintained at 37°C in an atmosphere of 95% air and 5% CO_2_.

### Irradiation

Cells were irradiated using a gamma irradiator (Caesium-137 source) and allowed to recover at 37°C for varying time periods.

### Proteasome inhibitor treatments

Cells were treated with proteasome inhibitor MG-132 and ALLN at final concentrations of 20 µM and 100 µM in DMSO (0.1%) respectively. Control cells were treated with 0.1% DMSO.

### Synchronization

Cells were synchronized in S-phase with double thymidine and in G2/M phase by thymidine-nocodazole treatment as described previously [34]. For cell cycle progression measured by FACS analysis, cells were fixed in 70% ethanol and stained with propidium iodide (Sigma).

### Western blotting and immunoprecipitation

Cells were pelleted, washed three times in 1×PBS, and lysed with lysis buffer (Tris 50 mM pH 7.4, NaCl 2.5 M, EDTA 5 mM, Triton X-100 0.1%) containing 1× complete protease inhibitors cocktail (Roche, Indianapolis, IN, USA). Total protein (30–50 µg) was heated 5 min at 90°C in 4× sample buffer (Invitrogen). Denatured samples were separated on NuPAGE 4–12% Bis-Tris gel or 3∼8% Tris-Acetate gel (Invitrogen), transferred to nitrocellulose membrane, and probed with the indicated primary antibody. Immunocomplexes were detected by incubation with peroxidase-conjugated secondary antibody and ECL chemiluminescence detection (Amersham). For *in vivo* BRCA1 ubiquitylation, cells were lysed in RIPA buffer (50 mM Tris-HCl pH 7.4, 150 mM NaCl,1 mM PMSF, 1 mM EDTA, 1% Triton X-100, 1% Sodium deoxycholate, 0.1% SDS, 1× protease inhibitors cocktail (Roche)) and then BRCA1 protein was immunoprecipitated from 2 mg total cell extracts for 16 hours at 4°C using anti-BRCA1 antibody. The antibody was captured by incubation using protein A/G agarose beads (PIERCE) for 2 hour at 4°C. Beads were washed 3 times in 1 ml ice-cold RIPA buffer followed by 2 times in 1 ml ice-cold PBS and heated for 10 minutes at 95°C in 2× Laemmli sample buffer. Immunoprecipitated proteins were analyzed by Western blotting with anti-ubiquitin antibody.

### RT-PCR

RNA was extracted using the TRIzol (Invitrogen) and was reverse transcribed using random hexamers as reaction primers. Quantitative assessments of cDNA amplification for BRCA1 [35] and the internal reference gene 18S were performed by a fluorescence-based real-time detection method (Biorad, Munchen, Germany) and the SYBR Green SuperMIX (Biorad). The oligonucleotides used are described previously (35). Polymerase chain reaction used consisted of 3 min at 95°C, followed by 30 cycles at 95°C for 15 s and 60°C for 1 min. To assure the amplicon specificity for each primer set, the PCR products were then subjected to a melting curve analysis. For each PCR, a standard curve was produced, using four consecutive 1∶10 dilutions of a positive sample. All samples were run in triplicate.

### *In vitro* degradation

Cell extracts were prepared from γ irradiation treated and untreated cells as described previously [34], [36]. ^35^S-labeled HA-tagged human wild-type BRCA1 and mutant BRCA1 proteins were synthesized by the TNT reticulocyte lysate system (Promega). Reaction mixtures containing 10 ng of ^35^S-labeled protein, 1.25 mg/ml ubiquitin (Sigma), 0.1 mg/ml cycloheximide (Sigma) and an energy regeneration system were incubated at room temperature. Aliquots were removed at indicated time points and reactions were terminated by the addition of SDS sample buffer. Samples were analyzed by 10% SDS-PAGE.

### Colony formation assay

Long-term survival of HeLa cells was determined in a clonogenic assay. Briefly, after treatment, cells were plated (50 cells/well) in 6 well plates. After 10 to 12 days in culture, colonies were exposed to staining solution containing 0.25% crystal violet and 10% formalin (35% v/v) in 80% methanol for 30 min, washed with water, and counted.

### Apoptosis analysis

After treatment, cells were harvested and washed once with PBS. PARP cleavage, Annexin V-FITC staining and flow cytometric analysis were used to assess apoptosis. PARP cleavage was detected by Western blotting. Annexin V-positive or sub-G1 peak cells were defined as a percentage of apoptotic cells.

## Results

### BRCA1 protein levels are drastically altered in response to γ irradiation

**Figure 1:**
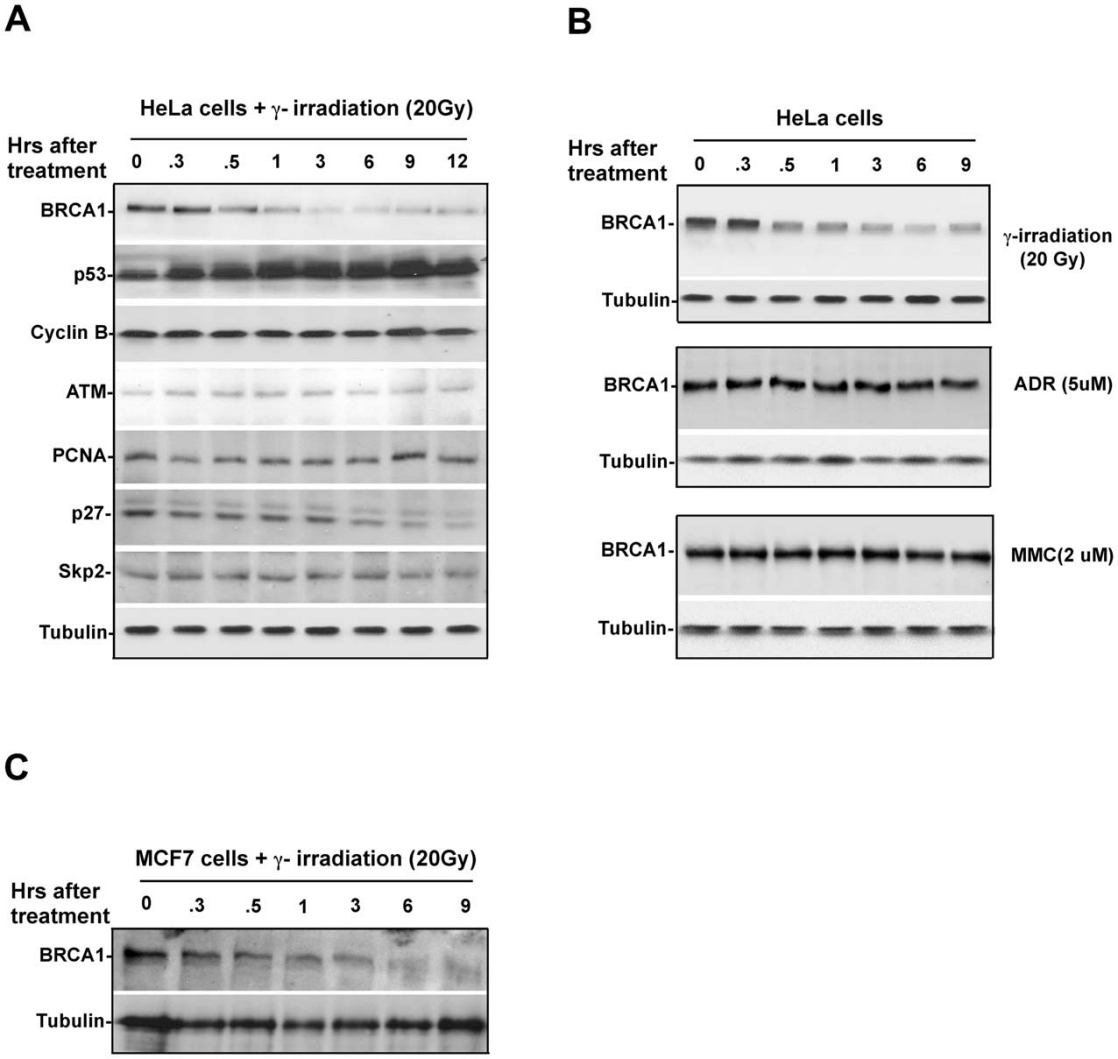
BRCA1 protein levels are altered by γ irradiation. **A**. BRCA1 protein levels rapidly drop in response to γ irradiation in HeLa cells. Cells were collected at different time points followed by exposure to γ irradiation. BRCA1 protein levels were monitored by immunoblotting using antibody against BRCA1. **B**. BRCA1 protein levels are altered by γ irradiation but remain stable in response to other DNA damaging agents, including ADR and MMS. C. γ irradiation at 20 Gy induces rapid decrease of BRCA1 protein levels in human breast cancer cell MCF7.

To study the role of UPS in genomic integrity, we have established a system to screen γ irradiation-induced degraded protein [34], [37]. Surprisingly, we found that BRCA1, a key protein in DNA damage response, was degraded in response to relatively high dose of γ irradiation (20 Gy) but not in response to low dose (5 Gy, Figure S1). To further validate this observation, HeLa cells were treated with 20 Gy γ irradiation and a time course evaluation of BRCA1 protein expression was examined. As shown in Figure 1A, the decrease in BRCA1 protein expression began about 30 minutes after irradiation and undetectable BRCA1 protein levels lasted for over 12 hours, while other examined proteins including cyclin B, ATM, PCNA and Skp2 were stable. We also tested the stability of BRCA1 protein in response to other genotoxic stress such as ADR (Adriamycin) and MMS (Mitomycin). As shown in Figure 1B, BRCA1 was rapidly degraded in response to γ irradiation, while no detectable alteration of BRCA1 was observed in the presence of ADR or MMS. To understand the change in stability of BRCA1 in response to the γ irradiation at different stages of the cell cycle, we examined the kinetics of BRCA1 protein levels in MCF7 cells after exposure to γ irradiation. As shown in Figure 1C, BRCA1 was drastically degraded in response to γ irradiation in MCF7 cells, suggesting that γ irradiation-induced BRCA1 degradation is not a cell type specific event.

### γ irradiation-induced BRCA1 degradation is mediated by the UPS

**Figure 2:**
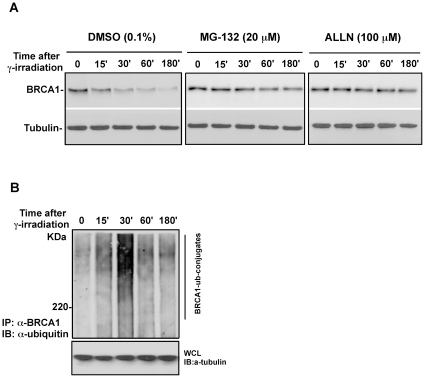
γ irradiation-induced degradation of BRCA1 is mediated by the ubiquitin-proteasomal pathway. **A**. γ irradiation-induced degradation of BRCA1 is blocked by proteasome inhibitor MG-132 or ALLN. HeLa S3 cells were preincubated with DMSO (vehicle control), MG-132 and ALLN for 20 minutes followed by exposure to γ irradiation. Cells were collected at indicated time points. BRCA1 protein levels were detected by immunoblotting. BRCA1 mRNA was monitored using RT-PCR. **B**. BRCA1 is ubiquitylated prior to degradation following exposure to γ irradiation. HeLa cells were harvested at different time points after exposure to γ irradiation. Cell pellet was lysed with denature-lysis buffer. Ubiquitin-conjugated BRCA1 was pulled down by antibody against BRCA1 and further detected by immunoblotting using antibody against ubiquitin.

To ask whether γ irradiation-induced BRCA1 turnover is mediated by the UPS, we examined the possible change in transcriptional regulation of BRCA1 in response to severe γ irradiation. As shown in Figure 2A, no significant alteration of BRCA1 mRNA was detected after γ irradiation, while BRCA1 protein levels dropped dramatically, suggesting that the drop in BRCA1 protein levels caused by γ irradiation was due to protein turnover. To further confirm that γ irradiation-induced BRCA1 turnover is mediated by the UPS, we examined the effect of proteasomal inhibitor on γ irradiation-induced BRCA1 degradation. As shown in Figure 2A, γ irradiation-induced BRCA1 degradation was largely blocked by MG132 or ALLN suggesting γ irradiation-induced BRCA1 degradation is through the proteasomal pathway, consistent with previous reports [17], [19]. To test whether BRCA1 was conjugated to ubiquitin molecules induced by γ irradiation, we carried out immunoprecipitation of BRCA1 coupled with immunoblotting using anti-ubiquitin antibody. As shown in Figure 2B, ubiquitin-conjugated BRCA1 was obviously detected at 15 minutes and peaked 30 minutes after γ irradiation. Taken together, our results suggest that BRCA1 is degraded in an ubiquitin-proteasomal dependent manner in response γ irradiation.

### BRCA1 protein stability is sensitive in response to γ irradiation during S and G2/M of the cell cycle

**Figure 3:**
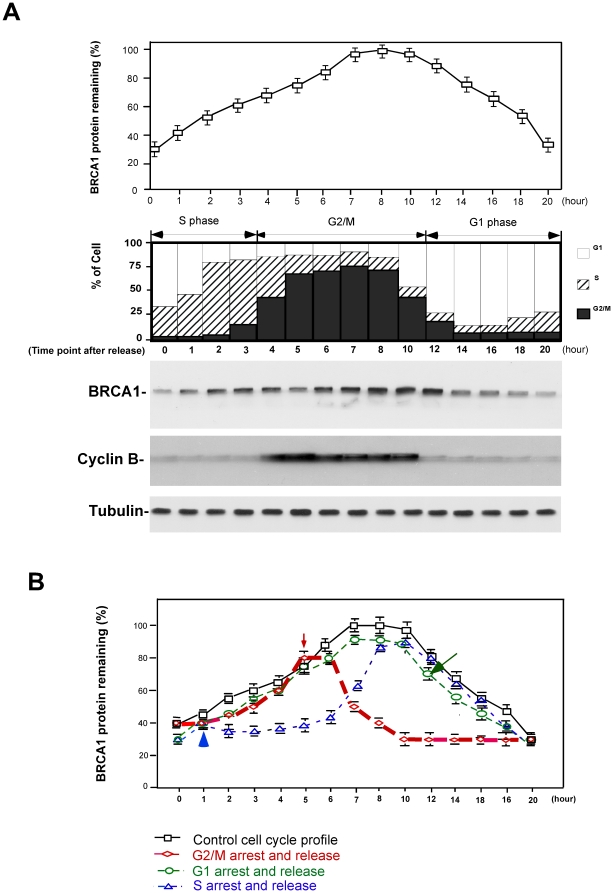
Different sensitivities of BRCA1 protein to γ irradiation at different stages of the cell cycle. **A**. BRCA1 protein fluctuates during the cell cycle. HeLa cells were synchronized with double thymidine and released. Cells were collected at different time points and BRCA1 protein levels were estimated by immunoblotting. **B**. BRCA1 protein levels are sensitive to γ irradiation at G2/M and S phase of the cell cycle. HeLa cells were synchronized at different stages followed by exposure to γ irradiation. γ irradiation-treated cells were subsequently released and collected at different time points for detection of BRCA1 protein levels.

BRCA1 has been described as a multiple-functional protein, which plays an important role in cell cycle control and apoptosis besides its function in genomic integrity [38]. A recent study has demonstrated that BRCA1 protein levels fluctuate during the cell cycle [19]. To test the response of BRCA1 protein levels to γ irradiation at different stages of the cell cycle, we measured the kinetics of BRCA1 protein levels during the cell cycle by cellular synchronization coupled with immunoblotting. As shown in Figure 3A, BRCA1 protein accumulated in G2/M and was maintained at relatively low levels in G1 and S phase, which is consistent with previous observation [19]. We next synchronized cells at different stages and then treated the synchronized cells with γ irradiation at G2/M, G1 and S phase and monitored the kinetics of BRCA1 protein levels. Comparing the pattern of BRCA1 oscillation during the cell cycle, we noticed that exposure of the synchronized cells to γ irradiation at G2/M and S phase caused dramatic alteration of the BRCA1 protein levels, while no significant change was observed for the cells treated at G1 phase (Figure 3B). Taken together, these results suggest that BRCA1 protein levels are sensitive to γ irradiation at G2/M and S phase during the cell cycle.

### Mapping the domain of BRCA1 mediating the γ irradiation-induced BRCA1 degradation

**Figure 4:**
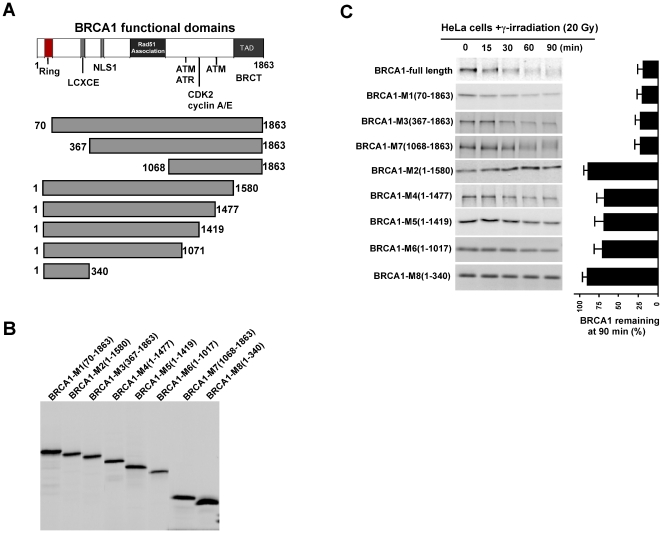
Mapping of functional domains mediating γ irradiation-induced BRCA1 degradation. **A**. Schematic presentation of BRCA1 functional domains, including Ring, LCXCE, NLS Rad51 binding, ATM/ATR, CDK2/cyclin A,E, TAD domains. **B**. Evaluation of the expression of BRCA1 mutants. **C**. Protein stability for BRCA1 mutants in response to γ irradiation was estimated using a cell-free protein degradation assay. HeLa cell lysate was prepared from the cells exposed to γ irradiation. ^35^S labeled and *in vitro* translated BRCA1 mutant proteins were subject to the functional lysate with supplements for protein degradation. Aliquot was collected at indicated time points and protein sample was resolved by SDS-PAGE. Protein stability for BRCA1 mutants in the presence of γ irradiation was reflected by autoradiography. Quantification was done with a Fuji PhosphorImager.

To determine the region of BRCA1 that confers the degradative response to γ irradiation, we generated a series of BRCA1 deletion mutants and examined the stability of these BRCA1 mutants using an *in vitro* protein degradation assay (Figure 4A and B) [34]. In this protein degradation assay, ^35^S-labeled *in vitro*-translated wild-type BRCA1 and its mutants were subjected to cell extracts prepared from γ irradiation-treated cells. Aliquots were then collected at different time points. Protein stability of the BRCA1 mutants was detected by SDS-PAGE and auto-radiography [34]. As shown in Figure 4C, all mutants missing the C-terminus were quite stable in response to γ irradiation, while the wild-type and all N-terminal mutants were degraded in the extract prepared from the cells treated with γ irradiation. This result suggests that the C-terminal region is important in facilitating the γ irradiation-induced BRCA1 degradation.

### BRCA1 degradation is correlated with the cellular survival and apoptosis after γ irradiation

**Figure 5:**
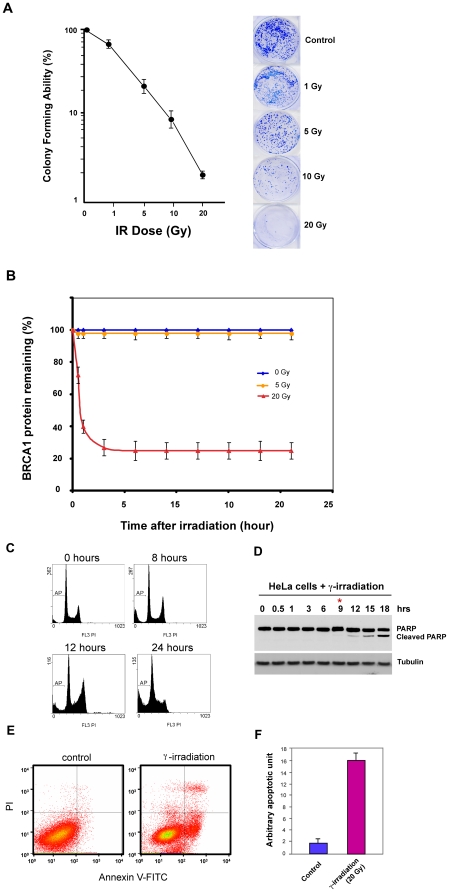
γ irradiation induces apoptosis in HeLa S3 cells. **A**. Clonogenic cell survival curves of HeLa cells after exposure to γ irradiation. **B**. Quantification of BRCA1 protein levels in response to γ irradiation at different doses. BRCA1 protein levels dropped significantly at high doses, while it remained stable after exposure to γ irradiation at low dose. Approximately 10,000 cells were plated on Petri dishes. Cells were exposed to γ irradiation and incubated in fresh medium for 10–14 days. Colonies were fixed and stained with a modified Giemsa solution (Fluka). Colonies of 50 or more cells were counted and data were expressed as percentage of colony formation relative to untreated controls. **C and D**. γ irradiation induces HeLa S3 cells apoptosis in a time-dependent manner. Hela S3 cells were irradiated at 20 Gy and harvested at indicated time points. Apoptosis was indicated as sub-G1 peak by FACS (A) and PARP cleavage by immunoblotting. **E** and **F**. Quantification of apoptosis induced by γ irradiation in HeLa S3 cell using Annexin V staining. Cells were treated with γ irradiation. Cells were stained with Annexin V and PI after 24 hours exposure to γ irradiation. The apoptotic cells (Annexin V+/PI −) were subsequently quantified by FACS. Results are mean ± s.d. of three independent experiments.

Our preliminary study has suggested that BRCA1 protein stability is dependent on the dosage γ irradiation. To explore the possible physiological relevance for acute γ irradiation-induced BRCA1 degradation, we initially tested the cellular survival in response to γ irradiation using a clonogenic cell survival analysis [39]. As shown in Figure 5A and B, γ irradiation affected the cell survival in a dosage dependent manner. Two weeks after irradiation, cellular colonies significantly dropped at a dosage of 5 or 10 Gy, while almost no colonies were found on the plate treated with 20 Gy, suggesting that irradiating cells with severe doses of γ irradiation induces cell death (Figure 5A and B).

To dissect the effect of γ irradiation on induction of cell apoptosis and further examine the correlation between the BRCA1 degradation and apoptosis, we tested the effect of γ irradiation on cell apoptosis using three different assays, including measuring cell cycle profile (sub-G1 peak), PARP cleavage and Annexin-V staining [36]. As shown in Figure 5C–F, exposure of cell to γ irradiation(20 Gy) significantly induced cellular apoptosis as demonstrated by the measurement of sub-G1 peak, PARP cleavage as well as the results from Annexin-V staining. Given that acute γ irradiation induces rapid BRAC1 degradation, our result suggests that the acute γ irradiation induced rapid BRCA1 turnover may be correlated with cellular apoptosis after γ irradiation.

### BRCA1 modulates the cellular susceptibility for apoptosis induced by γ irradiation

**Figure 6:**
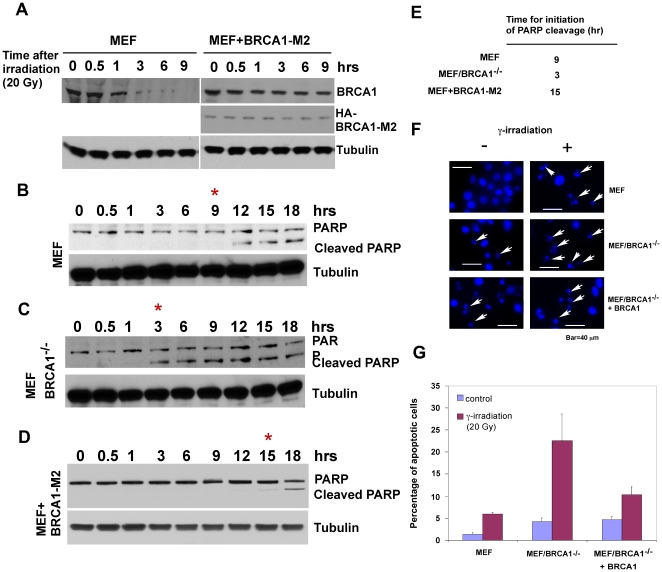
BRCA1 is important in modulating the onset of apoptosis in the presence of γ irradiation. **A**. BRCA1 is degraded in response to γ irradiation in MEF cells. **B**. Data based on measurements of PARP cleavage showed that initiation of γ irradiation-induced apoptosis (at 20 Gy) was detected about nine hours after exposure to γ irradiation. **C**. Loss of BRCA1 significantly enhanced the onset of apoptosis as reflected by an approximate six hours upshift for PARP cleavage. **D**. Expression of a non-degradable BRCA1 in MEF cells delayed the onset of γ irradiation-induced apoptosis. **E**. Summary of time for activation of apoptosis under different background of BRCA1. **F**. Apoptosis was visualized by fluorescence microscopy. γ irradiation treated cells were fixed and stained with DAPI and nuclear morphology was observed. Arrow indicates apoptotic cells. **G**. Quantification of γ irradiation-induced apoptosis in MEF, MEF/BRCA1^−/−^, and MEF/BRCA1^−/−^ + BRCA1 cells. Cells were stained with Annexin V and PI and apoptotic cells (Annexin V+/PI −) were quantified by FACS. Results are mean ± s.d. of three independent experiments (G).

To analyze the biological relevance of the γ irradiation mediated decrease in BRCA1, we examined the effect of BRCA1 protein levels on cellular susceptibility for the onset of apoptosis. We compared the susceptibility of apoptosis in mouse embryonic fibroblast cells and their corresponding BRCA1-null cells. Consistent with the result for HeLa cells, we observed that levels of BRCA1 significantly decreased in response to γ irradiation at 20 Gy in MEF cells (Figure 6A). Using PARP cleavage as a readout, we estimated the onset of apoptosis between wild-type and BRCA1-null MEF cells. As shown in Figure 6B and C, cleaved PARP was observed in wild-type cells 9 hours after exposure to γ irradiation, while loss of BRCA1 significantly enhanced an early PARP cleavage at 3 hours after γ irradiation in BRCA1- null cells. Our molecular mapping results showed that a loss of C-terminal BRCT domain could stabilize BRCA1 in the presence of γ irradiation (Figure 4C). To test the effect of BRCA1 stabilization on the γ irradiation-induced apoptosis, we performed an interference experiment by transfecting a non-degradable BRCA1 (1–1580) mutant. As shown in Figure 6D and E, overexpression of non-degradable BRCA1 significantly delayed the initiation of apoptosis. Similar to this observation, the overexpression of full-length BRCA1 delayed the onset of apoptosis induced by γ irradiation as demonstrated by nuclei condensation and Annexin V staining (Figure 6F and G). These results suggest that BRCA1 plays an important role in modulating the onset of apoptosis induced γ irradiation.

## Discussion

The critical role for BRCA1 has been demonstrated in multiple cellular processes, including cell cycle regulation, genomic integrity, development and apoptosis [40]. In the past decade, intense efforts have been made to address the mechanism by which BRCA1 is regulated, especially its phosphorylation by the checkpoint kinase and transcriptional regulation. In this study, we report that severe γ irradiation induces rapid BRCA1 degradation. Our findings suggest that the degradation of BRCA1 by acute γ irradiation could be the one of mechanisms for initiating apoptosis. Furthermore, our results demonstrate that UPS plays an important role in mediating γ irradiation-induced BRCA1 turnover, which in turn facilitates apoptosis.

### BRCA1 may guard the cell from genotoxic stress by preserving the cell from apoptosis

It has been reported that BRCA1 is associated with a large protein complex named the BRCA1-associated genome surveillance complex (BASC) that includes DNA damage detection molecules (e.g., ATM), DNA repair proteins (e.g., RAD50, MRE11, NBS1 and BLM), and mismatch repair proteins (e.g., MLH1, MSH2, and MSH6) [41]. These associations allow BRCA1 to participate in all processes required for DNA repair, such as detection of DNA damage, prevention of abnormal DNA replication, and replacement of damaged nucleotides. BRCA1 has also been reported to be involved in various DNA repair pathways including homologous recombination repair and nonhomologous end joining after ionizing radiation [42], [43] transcription-coupled repair of oxidative-induced DNA damage [44] and global genomic repair of UV-induced cyclobutane pyrimidine dimmers [45]. Given its important roles in DNA repair, it makes intuitive sense that BRCA1 could enhance cell survival after DNA damage by preventing apoptosis [46], [47]. For instance, reduction of BRCA1 expression results in increased sensitivity to the DNA damaging agents [48], whereas overexpression of BRCA1 increases resistance to the DNA damaging agents [49]. Moreover, reconstitution of BRCA1 in the BRCA1 mutant HCC1937 breast cancer line or BRCA1-null UWB1.289 ovarian cancer cell line resulted in a reduced sensitivity to the DNA damage chemotherapeutic agents or ionizing radiation [50], [51].

### BRCA1 destruction by UPS is required for the onset of γ irradiation-induced apoptosis

Besides the physiological relevance to genotoxic stress response, consideration must be given to situations when damaged DNA is not able to be repaired and thus the cell undergoes apoptosis to avoid the accumulation of mutated cells. Our findings suggest that BRCA1 may function as a “barrier”, mitigating the effects of genotoxic tress by preventing the onset of apoptosis. Removal of BRCA1 seems to permit the initiation of apoptosis induced by acute γ irradiation. In this present study, DSBs caused by 20 Gy IR cannot be repaired as evidenced by the persistence of γ-H2AX foci in MEF cells, which eventually lead cells to undergo apoptosis. Our data for BRCA1 analyses by loss of function or gain of function strongly suggest that BRCA1 abundance alters the cellular susceptibility to apoptosis. Coincidently, many well-established anti-apoptotic proteins such as Bcl-2s(50;51), XIAPs[52], [53], [54], and the inhibitor of κB (IκB) [55], [56], [57] have been shown to be tightly regulated by the UPS. Degradation of these anti-apoptotic proteins by the pro-apoptotic signaling activated UPS results in onset of apoptosis. Finding of BRCA1 degradation in modulating onset of apoptosis suggests that BRCA1 may be an apoptotic-resisting component.

### Mechanism by which BRCA1 is degraded

Protein stability of BRCA1 has been linked to the activity of proteasome, where supplementation of proteasomal inhibitors in the culture medium resulted in accumulation on BRCA1 [17]. Choudhury *et al*. demonstrated that BRCA1 protein levels fluctuate during cell cycle and alteration of BRCA1 is mediated by the UPS [19]. Biochemical dissection has revealed that BRCA1 can undergo ubiquitylation by a self-catalyzing mechanism via its ring domain [8], although we must note that the putative *in vivo* E3 ligase involved in regulating BRCA1 during the cell cycle remains unknown. Our data suggest the presence of an ubiquitin protein ligase mediating the genotoxic signaling for BRCA1 degradation. The results from mapping the degron suggest that the important region facilitating the BRCA1 degradation lies beyond the ring domain. The BRCT domain was thought to be important for the degradation event. This implies that the γ irradiation-induced BRCA1 ubiquitylation/degradation is not catalyzed by its auto-ubiquitylation, suggesting the presence of an additional E3 ligase that mediates BRCA1 degradation induced γ irradiation. The activity of such a ubiquitin protein ligase could be mediated by one or many different ligases involved in different stages of the cell cycle as reflected in BRCA1 oscillation during the cell cycle. To determine the potential E3 ligase involved in BRCA1 degradation, we have taken a biased approach by immunoblotting the BRCA1 IP complex purified from the cells exposed to γ irradiation with antibodies against several known E3 ligases, including SCF, APC, MDM2, Cul4A/DDB/ROC1 and COP1. We unfortunately have not presently identified any putative candidate by this strategy. To identify the E3 ligase governing the γ irradiation-induced BRCA1 degradation and further elucidate the mechanism of BRCA1 proteolysis, we have initiated a strategy using BRCA1-TAP purification by identifying BRCA1 complex in the presence of γ irradiation. This ongoing work could potentially address the mechanism by which BRCA1 is degraded by γ irradiation and provide additional insight as to how to therapeutically modulate BRCA1.

## Supporting Information

Figure S1 Alteration of BRCA1 protein levels after exposure to low dose of γ irradiation. HeLa cells were treated with low dose γ irradiation (5 Gy). Cells were collected at different time points followed by exposure to γ irradiation. BRCA1 protein levels were monitored by immunoblotting using antibody against BRCA1. β-actin was measured as loading control. No obvious alteration of BRCA1 protein levels was observed in response to low dose γ irradiation.
(5.64 MB TIF)
