# Supplementary figures and images for: Turnover of BRCA1 Involves in Radiation-Induced Apoptosis
Source: PLoS One. 2010 Dec 31;5(12):e14484. doi: 10.1371/journal.pone.0014484 (PMC3013096; doi:10.1371/journal.pone.0014484)

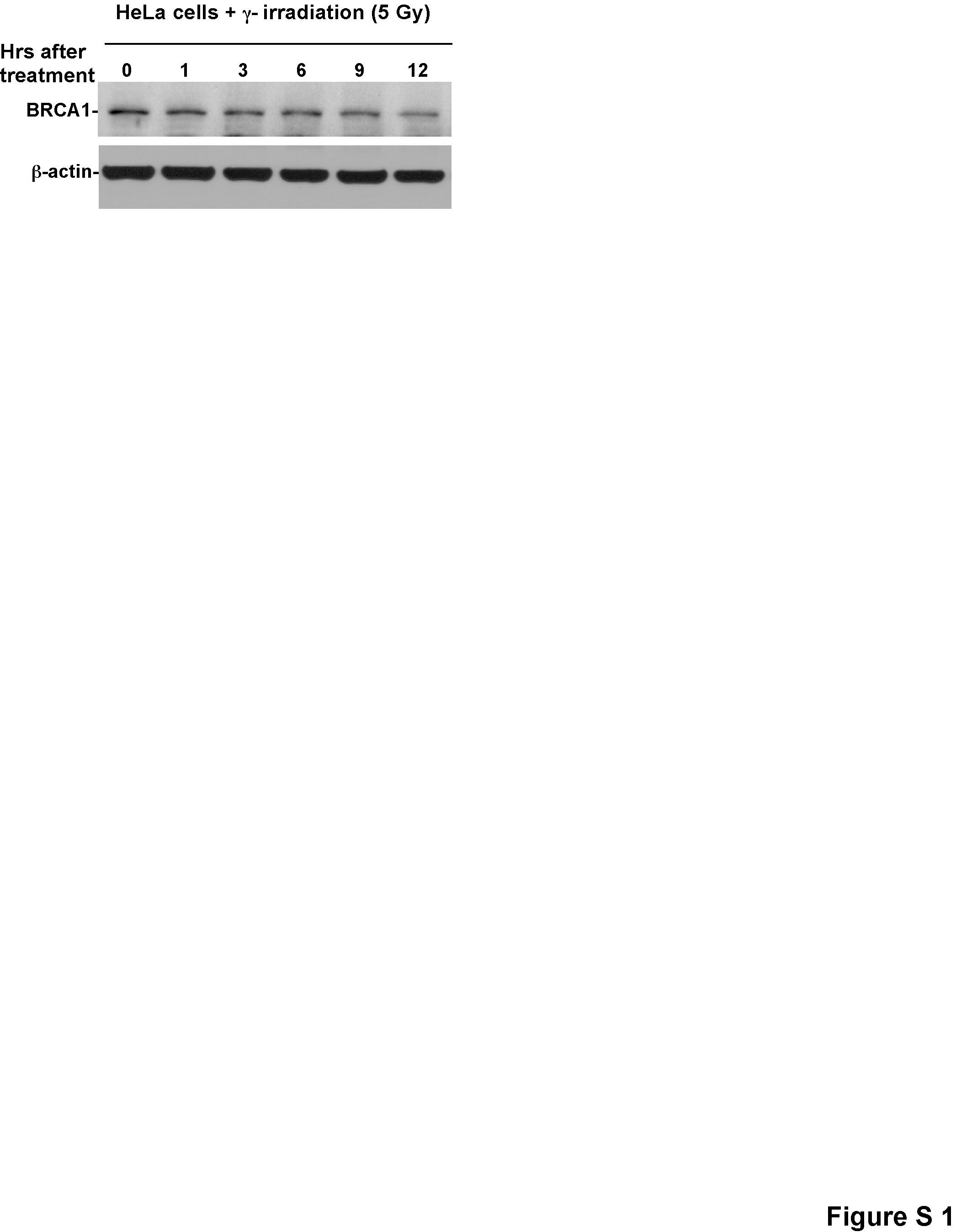

Supplement: Figure S1 — Alteration of BRCA1 protein levels after exposure to low dose of γ irradiation. HeLa cells were treated with low dose γ irradiation (5 Gy). Cells were collected at different time points followed by exposure to γ irradiation. BRCA1 protein levels were monitored by immunoblotting using antibody against BRCA1. β-actin was measured as loading control. No obvious alteration of BRCA1 protein levels was observed in response to low dose γ irradiation. (5.64 MB TIF) [file pone.0014484.s001.tif]
